# New Insights on CD8^+^ T Cells in Inflammatory Bowel Disease and Therapeutic Approaches

**DOI:** 10.3389/fimmu.2021.738762

**Published:** 2021-10-11

**Authors:** Rosaely Casalegno Garduño, Jan Däbritz

**Affiliations:** ^1^ Mucosal Immunology Group, Department of Pediatrics, Rostock University Medical Center, Rostock, Germany; ^2^ Center for Immunobiology, Blizard Institute, The Barts and the London School of Medicine and Dentistry, Queen Mary University, London, United Kingdom

**Keywords:** CD8^+^ Tc1, Tc17, T regs, TRM, Crohn´s disease, ulcerative colitis, IBD

## Abstract

CD8^+^ T cells are involved in the pathogenesis of inflammatory bowel disease (IBD), a complex multifactorial chronic disease. Here, we present an overview of the current research with the controversial findings of CD8^+^ T cell subsets and discuss some possible perspectives on their therapeutic value in IBD. Studies on the role of CD8^+^ T cells in IBD have contradictory outcomes, which might be related to the heterogeneity of the cells. Recent data suggest that cytotoxic CD8^+^ T cells (Tc1) and interleukin (IL) 17-producing CD8^+^ (Tc17) cells contribute to the pathogenesis of IBD. Moreover, subsets of regulatory CD8^+^ T cells are abundant at sites of inflammation and can exhibit pro-inflammatory features. Some subsets of tissue resident memory CD8^+^ T cells (Trm) might be immunosuppressant, whereas others might be pro-inflammatory. Lastly, exhausted T cells might indicate a positive outcome for patients. The function and plasticity of different subsets of CD8^+^ T cells in health and IBD remain to be further investigated in a challenging field due to the limited availability of mucosal samples and adequate controls.

## Introduction

Inflammatory bowel disease (IBD) affects millions worldwide (1). Its etiology is a combination of environmental factors, a defective immune system and an altered intestinal microbiota (dysbiosis) in genetically susceptible individuals (2, 3). IBD comprises two main conditions, namely Crohn´s disease and ulcerative colitis. Crohn´s disease is a chronic intestinal inflammation that can affect any part of the gastrointestinal tract from the mouth to the anus causing abdominal pain, weight loss and altered bowel pattern (4). In contrast, ulcerative colitis affects mainly the colon and the rectum and patients often present bloody diarrhea (3).

The main sites of inflammation in IBD are the intestines (4, 5), home to the largest density and greatest diversity of commensal microbiota (3, 6, 7). Containing the microbiota in its niche implies tolerance by the immune system in a complex process known as homeostatic immunity (8). Such homeostasis enables beneficial host–commensal relationships and it is imperative for the development of a healthy gut and, ultimately, the host survival (8–11). To acquire homeostatic immunity two major mechanisms regulate the interaction of the microbiota with its host. First, a balance must exist between both tolerance towards commensals and controlling of any potential pathogen (7) and second the mucosal barrier must be maintained. Supported by observations in patients with IBD, disruption of this delicate equilibrium is causative for inflammatory reactions in the digestive system (9, 12, 13). In addition, losing the integrity of the important mucosal barrier, a single layer of epithelial cells and a goblet-cell-produced mucus layer, allows luminal microbial products or even commensal bacteria to enter the subjacent lamina propria, thus triggering an inflammatory immune response (3, 8, 14). Subsequently, innate and adaptive immune cells are able to aberrantly infiltrate the mucosa exacerbating the inflammation in IBD patients (5, 6, 12). In this regard, an excessive activation of effector T cells and an altered T cell-mediated tolerance were identified as key players in the onset/course of the disease (6, 12).

The vast majority of mature T cells express either CD4 or CD8αβ glycoprotein, which has been used to divide them into 2 subpopulations, CD4^+^ or CD8^+^, respectively (13, 15). Both activated CD4^+^ and CD8^+^ T cells have been found in peripheral blood and intestinal mucosa of adult and pediatric IBD patients during inflammation (12, 16–18). While researchers have mainly focused on the association of CD4^+^ T cells, growing attention is now being given to the role of CD8^+^ T cells in IBD. New experiments have been designed to explore their heterogeneity and involvement in this life-lasting disease (19–21). This review article presents an overview of the current research with the controversial findings of CD8αβ^+^ T cell subsets and discusses some possible perspectives on their therapeutic value in IBD.

## CD8^+^ T Cells Contribute To The Pathogenesis Of IBD

**Figure 1:**
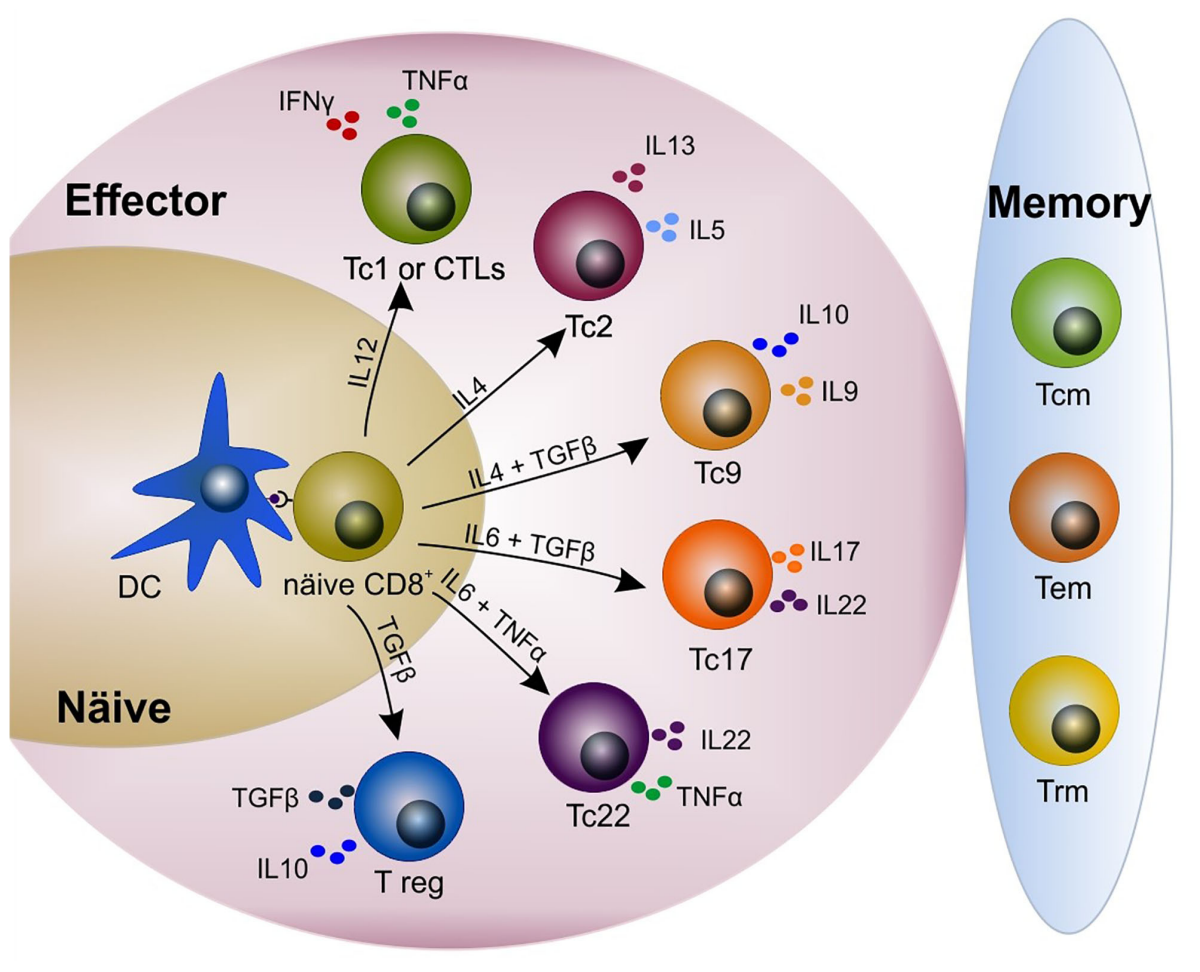
Heterogeneity in the CD8^+^ T cell pool. Upon antigen presentation and cytokine release by dendritic cells (DCs), naïve CD8^+^ T cells differentiate into different subsets including cytotoxic/cytolytic (Tc1 or CTLs), Tc2, Tc9, Tc17, Tc22, and immunosuppressant T regulatory (T reg). CD8^+^ T cells produce different cytokines according to their phenotype. A few clones remain and constitute the memory compartment (*i.e.* central memory, effector memory, tissue-resident memory). Modified from Golubovskaya and Wu (15), Mittrücker et al. (22) and St. Paul and Ohashi (23). CTL, cytotoxic T lymphocyte; DC, dendritic cell; Tcm, central memory T cell; Tem, effector memory T cell; Trm, tissue-resident memory T cell.

**Figure 2:**
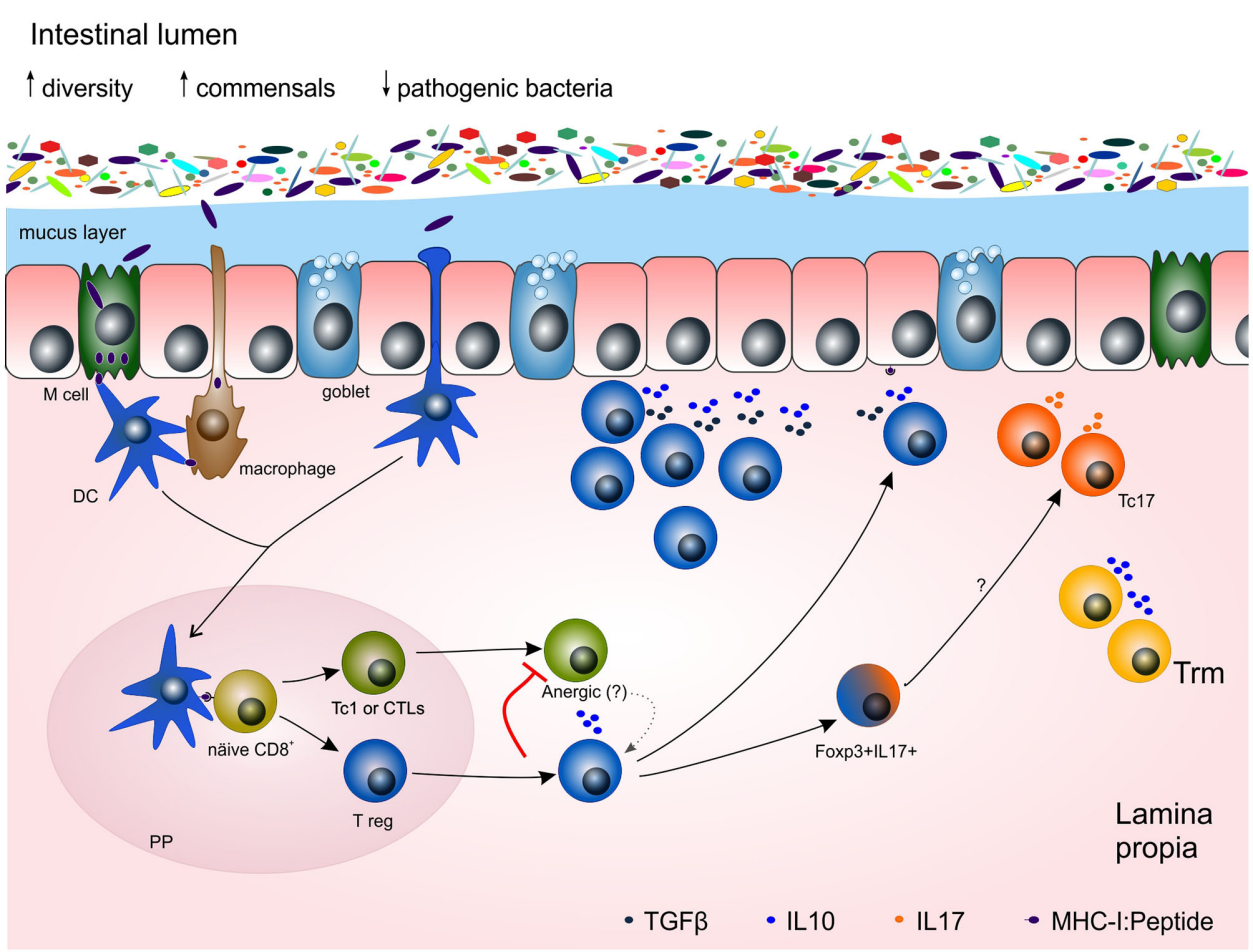
Potential mechanisms of the adaptive immune response towards gut microbiota in homeostasis and chronic inflammation. Under homeostasis, microbiota is restricted to the lumen of the gut by both an epithelial cell layer and a mucus layer, produced by goblet cells and full of antibacterial peptides. Dendritic cells (DCs), M cells and macrophages acquired antigens from the lumen. Antigen presenting cells (APCs) carrying those antigens migrate to Peyer´s patches (PP) where they present the antigens to naïve T cells to prime them. Regulatory CD8^+^ T cells are able to immunosuppress Tc1 by interleukin (e.g. IL10) release and cell-cell contact, leading Tc1 cells towards anergy, a non-responsive stage, or even re-directed towards a T reg phenotype. Some double positive Foxp3^+^ IL17^+^ cells might be in an intermediate stage towards Tc17, relevant cells for mucosal maintenance. Trm cells contribute to the homeostasis of the tissue by releasing IL10. DC, dendritic cell; IL, interleukin; PP, Peyer´s patch; Tc1 or CTL, cytotoxic T lymphocyte; Tc17, IL17-producing CD8^+^ T cells; T reg, CD8^+^ T regulatory cell.

T cells possess a T-cell receptor (TCR) that uniquely recognizes an epitope or antigen, giving the T cell its specificity. CD8^+^ T cells recognize the cognate antigen in the context of the major histocompatibility complex (MHC)-I presented by professional antigen presenting cells (APCs) such as macrophages and dendritic cells (DCs). Once APCs engage a naïve T cell in the lymph nodes, the T cell becomes activated and proliferates. Most of the clones will serve their purpose and, when no longer needed, die naturally in the contraction phase with just a few long-term survival cells remaining, constituting the memory cell compartment (13, 22) (**Figure 1**). Remarkably, tolerance against self-, harmless microbiota- and food-antigens must be achieved in the gut. Under homeostatic conditions different cell populations such M cells, goblet cells, DCs and CX3CR1^+^ macrophages transport luminal microbiota antigens into intestinal tissue (24, 25). CX3CR1^+^ macrophages acquire food- and microbiota-antigens by extending their protrusions into the lumen (25). Self-antigens from apoptotic intestinal epithelial cells (IECs) are sampled as well (24, 26). Antigens are then transferred to DCs which in turn present them to naïve T cells in the draining mesenteric lymph nodes and Peyer’s patches. The result of this DC-T cell interaction is to induce immunosuppressant regulatory T cells (T regs) (25, 26) (**Figure 2**) to maintain homeostasis. This delicate balance could already be impaired in patients with IBD. A point mutation in the *cx3cr1* gene has been observed in Cronh´s disease patients that leads to impaired immune responses (27). Complete abrogation of CX3CR1 on colonic macrophages results in a failure to support the expansion of lamina propria CD4^+^ T regs (1, 28). How this mutation affects CD8^+^ subsets remains open.

Although the notion of the involvement of CD8^+^ T cells in IBD has been long considered and published (12, 17), new attention has been given in more recent years (for summary of articles see **Table 1**). Throughout the years, the role of CD8^+^ T cells in IBD has been controversial, with some reports indicating anti-colitogenic properties (11, 42–44) with others showing their contribution to tissue inflammation (5, 12, 16–18, 20, 31, 36, 39, 45–47). Such ambivalent findings might be explained, at least partially, by the source from where CD8^+^ T cells were obtained (e.g. peripheral blood mononuclear cell -PBMC-, intestinal intraepithelial, lamina propria; **Table 1**). Another plausible explanation relies on the intrinsic differences of CD8^+^ T cell subsets. CD8^+^ T cells are in fact very broad in phenotype and function (19, 21–23) (**Figure 1** and **Table 1**). There are the well documented conventional cytotoxic CD8^+^ T cells (Tc1, also known as cytotoxic/cytolytic T lymphocytes, CTLs), as well as CD8^+^ regulatory T cells (CD8^+^ T reg) (48, 49), and many others including type II-cytokine-producer CD8^+^ (Tc2), interleukin (IL) 9^+^ CD8^+^ (Tc9), IL17^+^ CD8^+^ (Tc17) and IL22^+^ CD8^+^ (Tc22) (23). CD8^+^ T cells acquire different phenotypes depending on cytokine stimulation, co-stimulatory molecules as well as the strength on the TCR/antigen engagement (22, 23) (**Figure 1**). Highly heterogenic CD8^+^ T cell populations have been identified in the colon of IBD patients (19, 21). They not only have different phenotypes, but presumably, different functions (19, 21). Furthermore, transcriptional profiling studies on peripheral CD8^+^ T cells have shown their intrinsic differences among age, gender and inflammation status of IBD patients (29). Among adult IBD patients two clear expression signatures can be recognized, one that correlates to a mild outcome, and another one correlating to a severe one (e.g. upregulation of genes associated to antigen-dependent T cell activation such as signaling by TCR, IL-2, IL-7 and CD28 co-stimulation) (5). However, such signatures could not be corroborated in a different cohort analysis by Gasparetto et al. (29). This discrepancy reflects the complexity of IBD and the intrinsic differences among patients due to their clinical course, age, treatment, smoking status, gender, allergies, microbiota, etc. Similarly, pediatric IBD patients showed no clustering of expression signatures and CD8^+^ T cell transcription and DNA methylation profiles do not correlate to the clinical outcome of the pediatric IBD cohort (29).

**Table 1:** The contribution of CD8^+^ T cells in IBD.

Ref.	Key findings	Disease/disease status	CD8^+^ T cell origin	Experimental Settings
Gasparetto et al., 2021 (29)	Transcriptional signature and DNA methylation profiles of circulating CD8^+^ T cells from pediatric patients with active IBD does not correlate to clinical outcome	Pediatric active CD and UC	PBMCs	Genome transcript analysis of magnetic-sorted CD8^+^
Jaeger et al., 2021 (30)	Intraepithelial and lamina propria compartments harbor similar CD8^+^ T cell subsets. Circulating and tissue resident CD8^+^ T subsets were found in the intraepithelial compartment. CD8^+^ T cells were increased in non-inflamed LP of CD, whereas CD8^+^ T cells were decreased in the intraepithelium at sites of inflammation. KLRG1^+^ CD8^+^ T frequencies showed no difference between patients and CD patients.	Severe CD	Inflamed and non-inflamed terminal ileum biopsies	CyTOF and scRNA-seq on CD45^+^ IEL and LP cells. Cluster analysis on CD8^+^ cells
Boland et al., 2020 (19)	Single cell atlas of colonic CD8^+^ T cells from UC patients revealed highly heterogeneic populations. CD8^+^ Trm might be involved in the development of UC.	Active UC	PBMCs and rectal biopsies	scRNA-seq on CD45^+^ cells. Cluster analysis on CD8^+^ cells
Bottois et al., 2020 (20)	High numbers of CD103^+^ CD8^+^ Trm were found in the mucosa of CD patients and controls, but those from patients had a more prominent Th17 profile. KLRG1^+^ CD8^+^ Trm cells were increased in inflammatory conditions. In Crohn´s disease patients the CD103^+^ CD8^+^ T cells might alert the effector KLRG1^+^ CD8^+^ T subset.	Active CD	PBMC as well as inflamed and non-inflamed ileum biopsies	CD5^+^ magnetic and Aria sorted
Bruckner et al., 2020 (31)	Pro-inflammatory TNFα^+^ CD8^+^ T cells play a role on the development of fistulas in CD patients.	CD with fistula	Biopsies from acute inflamed fistulas	FACS on CD8^+^ cells
Corridoni et al., 2020 (21)	Single cell atlas of colonic CD8^+^ T cells from biopsies of UC patients. Informative platform for future functional studies.	Active UC	Inflamed colonic biopsies	scRNA-seq on CD3^+^ CD8^+^ cells
Libera et al., 2020 (32)	Mucosal-derived CD39^+^ CD8^+^ T cell frequency was decreased in IBD patients compared to healthy controls	Active CD and UC	PBMCs and colonic biopsies	FC analysis on CD39^+^ CD8^+^ cells
Noble et al., 2020 (33)	Circulating CD8^+^ memory T cells from HDs respond to intestinal bacteria derived antigens. Reduced numbers of mucosal CD8^+^ Trm were observed in IBD patients compared to controls.	CD and UC	Non-inflamed colonic biopsies and PBMC	FC analysis on CD103^+^ Runx3^+^ CD8^+^
Huang et al., 2019 (34)	Colonic *GZMK^+^* (granzyme K) CD8^+^ Tem cells were clonally expanded in a pediatric IBD cohort with active disease. Furthermore, colonic *ITGAE* (CD103) CD8^+^ Trm and *ENTPD1* (CD39) CD8^+^ Trm cells were decreased in pediatric IBD compared to controls.	Pediatric active CD and UC	Colonic biopsies	scRNA-seq on CD45^+^ cells. Cluster analysis on CD8^+^ cells
Rabe et al., 2019 (18)	Pediatric ulcerative colitis patients had higher levels of activated HLA.DR^+^ β1-integrin^+^ CD8^+^ T cells in the periphery and that correlated positively to systemic and mucosal inflammation biomarkers. Pediatric Crohn´s disease patients showed equal levels of activated HLA.DR^+^ β1-integrin^+^ CD8^+^ T cells to controls, but an increase on the CD23^+^ B cell population.	Newly diagnosed pediatric CD and UC	Blood	FC analysis on CD8^+^
Roosenboom et al., 2019 (35)	Decreased percentage of CD103^+^ CD8^+^ T cells in the inflamed ileum and colon of IBD patients compared to controls. CD103^+^ CD8^+^ T cell frequencies were comparable between IBD patients in remission and controls.	Active CD and UC (including follow-up)	Biopsies from inflamed colon and ileum	FC analysis on CD103^+^ CD8^+^
Smillie et al., 2019 (36)	Single cell atlas of colonic biopsies of UC patients revealed Tc17 subset as expanded, and major source of IL17, in inflamed tissue.	Active UC	Inflamed colonic biopsies	scRNA-seq on all cells. Cluster analysis on CD8^+^ cells
Zundler et al., 2019 (37)	Lamina propria CD103^+^ CD69^+^ CD8^+^ Trm cells were increased in inflamed mucosa of IBD patients compared to controls.	Active CD and UC	Colonic biopsies	FC analysis on CD103^+^ CD69^+^ CD8^+^
Smids et al., 2018 (38)	CD103^+^ CD8^+^ Trm cells were decreased in inflamed tissue of IBD compared to non-inflamed biopsies and controls. Furthermore, CD8^+^ Tcm were increased in inflamed intestine biopsies of UC patients with active disease, whereas CD8^+^ Tem frequency was decreased compared to controls.	Active CD and UC	Biopsies of inflamed intestine and of follow-up endoscopies	FC analysis on CD8^+^
Boschetti et al., 2016 (39)	Circulating and mucosal GrB^+^ perforin^+^ CTLs were abundant in CD patients with recurrent disease, compared to endoscopic remission and controls.	Recurrent CD	PBMC and curative ileum biopsies	FC analysis on CD8^+^
Tom et al., 2016 (40)	The frequency of lamina propria CD8^+^ T regs was higher in IBD patients compared to controls. Lamina propria Tc17 prevalence was higher in UC patients compared to CD patients and controls. Circulating CD8^+^ T regs and Tc17 were increased in IBD patients compared to controls.	Active CD and UC	PBMC and inflamed mucosa biosies (LPMC)	FC analysis on CD8^+^
Funderburg et al., 2013 (16)	Activated IFNγ^+^ CD8^+^ cells were increased in the peripheral blood of IBD patients, and correlate to higher levels of inflammation markers in serum.	Active CD and UC	PBMCs	FC analysis on CD8^+^
Lee et al., 2011 (5)	Two distinctive CD8^+^ T cell expression signatures can be recognized in adult patients with active IBD, one that correlates to a mild outcome, and another one correlating to a severe one. CD8^+^ T cells might disrupt epithelial barrier playing an earlier role in the development of the disease.	Active CD and UC	PBMCs	CD8^+^ magnetic sorted
Brimnes et al., 2005 (41)	Regulatory activity of CD8^+^ T cells from lamina propria of healthy gut was observed *in vitro*, whereas CD8^+^ T cells from lamina propria of IBD patients showed no regulatory activity	CD and UC	LPMCs from colon	FACS on CD8^+^

CD8^+^ T cell subsets contribute to IBD in a different manner, some pools are pro-inflammatory whereas other are anti-inflammatory. The source where CD8 cells were obtained from might reflect different phenotypes, hence different functions of the cells. CD, Crohn’s disease; CyTOF, cytometry by time of flight; ENTPD1, ectonucleoside triphosphate diphosphohydrolase-1 (CD39-encoding gene); HDs, healthy donors; IEL, intraepithelial lymphocyte; ITGAE, integrin alpha E (CD103-encoding gene); FACS, fluorescence activated cell sorting; FC, flow cytometry; GrB, granzyme B; GZMK, granzyme K-encoding gene; LP, Lamina propria; LPMC, Lamina propria mononuclear cells; PBMCs, peripheral blood mononuclear cells; scRNA-seq, single cell RNA sequencing; Trm, resident memory T cells; UC, ulcerative colitis.

Interestingly, newly diagnosed and medication-free pediatric ulcerative colitis and Crohn´s disease patients differ in their T cell activation pattern. On one hand, pediatric ulcerative colitis patients had higher frequencies of activated HLA.DR^+^ β1-integrin^+^ CD8^+^ T cells in the periphery and that correlated positively to systemic and mucosal inflammation biomarkers (e.g. fecal calprotectin, lactoferrin, eosinophil cationic protein). On the other hand, pediatric Crohn´s disease patients showed equal levels of activated HLA.DR^+^ β1-integrin^+^ CD8^+^ T cells to controls, but an increase on the CD23^+^ B cell population (18). Given that both diseases are closely related and that precise diagnosis is difficult in some cases, patterns of lymphocyte subsets could be a tangible extra diagnostic tool, at least in pediatric IBD patients.

It is clear, however, that CD8^+^ T cells have an impact on IBD; question is, which CD8^+^ subset does what?

## IBD Could Be, In Part, Initiated And Further Developed By Activated Cytotoxic CD8^+^ T Cells (Tc1)

Tc1 cells are very efficient in killing tumor, virus-infected and bacteria-harboring cells (22). Primed Tc1 cells produce high amounts of pro-inflammatory molecules such as interferon gamma (IFNγ), tumor necrosis factors (TNFα), granzyme B (GrB) and perforin (23). On the other hand, animal studies showed pathogenic Tc1 cells associated with the initiation and/or development of colitis (46, 47). IBD patients, most remarkably ulcerative colitis patients, have increased numbers of activated IFNγ^+^ Tc1 cells in peripheral blood, correlating to higher levels of inflammation markers in serum (16). Moreover, Boschetti and colleagues found that circulating and mucosal GrB^+^ perforin^+^ Tc1 cells are abundant in Crohn’s disease patients with endoscopic recurrence, compared to endoscopic remission and controls (39). In addition, Bruckner et al. reported on the inflammatory effects of Tc1 cells on the mucosa of Crohn’s disease patients leading to fistulas (31), common ulcers in up to 50% of the patients. In fact, activated perforin^+^ Tc1 cells are increased in the affected intestinal mucosa of active stages of the disease course in IBD patients (12, 17).

The relevance of Tc1 cells for mucosal damage is supported by colitis-induced mouse models (46, 47). For instance, the hapten 2,4 dinitrobenzene sulfonate (DNBS)-induced colitis model used by Nancey and colleagues. In this model, immunocompetent mice sensitized with DNBS developed colitis after DNBS challenge. The damage of the mucosa was associated to hapten-specific Tc1 cells; since CD8^+^ T cells, but not CD4^+^ T cells, proliferated and produced IFNγ in the *in vitro* enzyme-linked immunospot (ELISpot) assay. Remarkably, colitic mice progressively recovered on their own from acute colitis and reached remission. Moreover, the *in vivo* anti-CD8 antibody depletion showed complete absence of colon inflammation. Additionally, a relapsing episode could be induced by a second DNBS challenge, clearly as a result of the pro-inflammatory effects of hapten-specific Tc1 cells. Thus, the DNBS-induced colitis model resembled the chronic pathology occurring in IBD characterized by episodes of flare up and remission (47).

**Figure 3:**
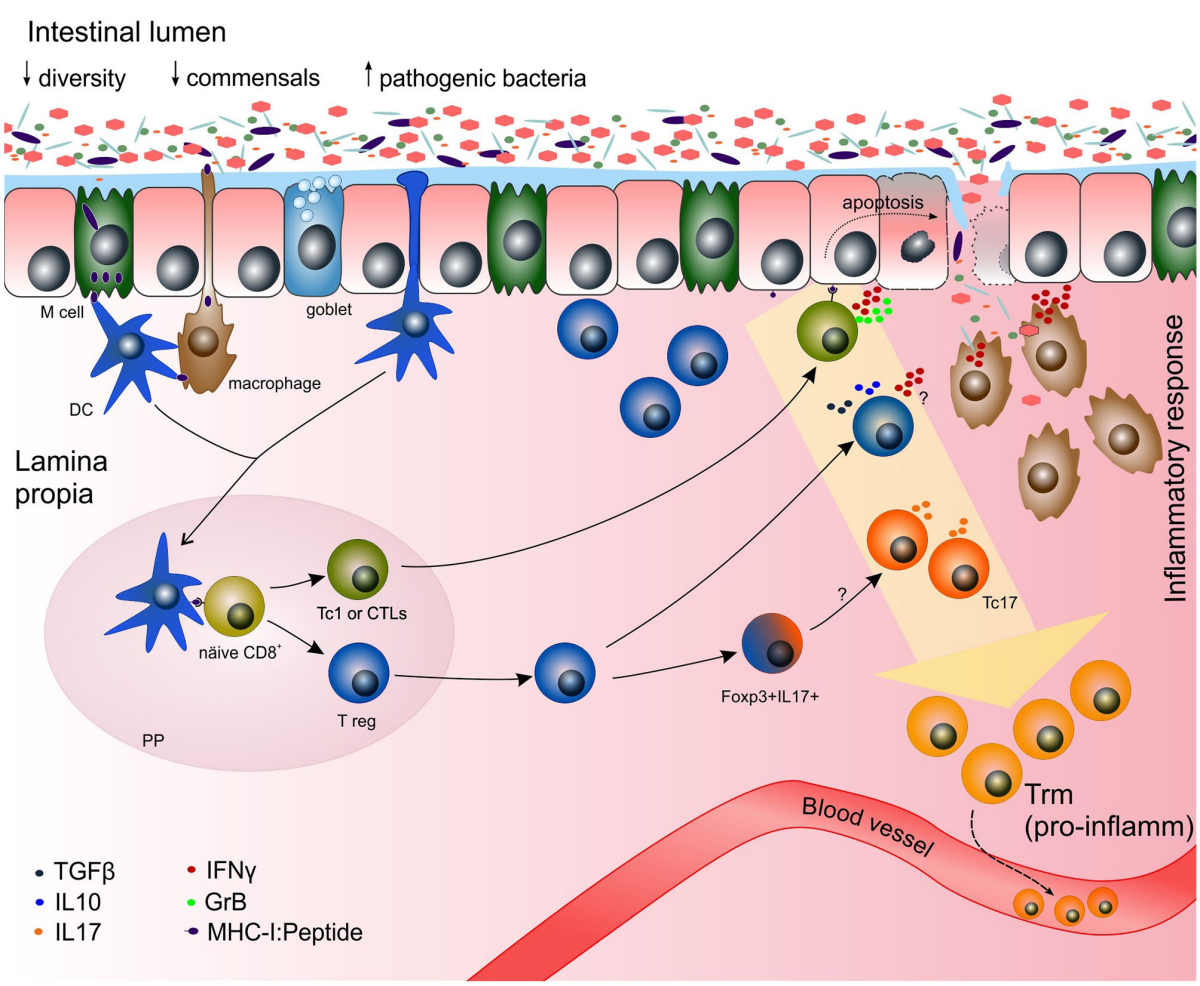
Potential mechanisms of the adaptive immune response towards gut microbiota in chronic inflammation. Increased numbers of pathogenic bacteria (e.g.* Clostridium difficile*, *Chlamydia pneumonia*, *Listeria monocytogenes*) have been reported in IBD patients (50). During chronic inflammation, functional cytotoxic CD8^+^ Tc1 cells might be predominantly generated. The disruption of the epithelial barrier might occur by the cytotoxic effect of commensal-specific Tc1 recognizing peptides derived from commensal bacteria on the MHC-I of epithelial cells. Once the epithelial barrier is broken, bacteria can pass freely to the lamina propria initiating an immune response by members of the innate immune system (e.g. macrophages). Due to the plasticity of the cells and a highly pro-inflammatory milieu, CD8^+^ T regs might be driven towards Tc1 or IFN^+^ T regs, making the damage even greater. Trm express pro-inflammatory genes in the context of inflammation. Those pro-inflammatory Trm might be derived from pro-inflammatory Tc1 and Tc17, and some clones might exit the tissue *via* the blood stream and initiate inflammation in other tissue outside the gastrointestinal tract. DC, dendritic cell; GrB, granzyme B; IFNγ, interferon gamma; IL, interleukin; MHC-I, major histocompatibility complex class I; PP, Peyer´s patch; Tc1 or CTL, cytotoxic T lymphocyte; Tc17, IL17-producing CD8^+^ T cells; TGFβ, transforming growth factor beta; T reg, CD8^+^ T regulatory cell.

Moreover, pro-inflammatory cytokines released by Tc1 cells might be involved in epithelial barrier disruption. These factors not only physically disrupt the barrier by inducing apoptosis to the epithelium (46, 47) (**Figure 3**), but also increase intestinal permeability (51) leading to loss of barrier function. For instance IFNγ and TNFα disturb IEC tight junctions resulting on a disruption of colon epithelium (52). Furthermore, *in vitro* studies have shown increased apoptosis of IECs due to chronic exposure of IFNγ (53, 54), that exacerbates under the synergistic effect of TNFα (53). Both IFNγ and TNFα are increased in the colon of colitis mouse models during flare-up (47, 53). Moreover, chronic intestinal inflammation decreased IEC proliferation in both animal models and IBD patients (53, 55) leading to an impaired barrier function.

Arguably, Tc1 cells specific to microbiota- or food-derived antigens are involved in the pathogenesis of IBD. Although microbiota-derived antigen specific CD8^+^ T cells are found in the periphery of both healthy individuals and IBD patients (33), they might not always be reactive in healthy individuals evidenced by the lack of inflammation (56). Speculatively, microbiota- and food-derived antigen specific Tc1, as well as autoreactive Tc1 cells, threaten homeostasis. One might assume that those Tc1 able to recognize harmless antigens might be tightly controlled in peripheral tolerance. Evidence suggests that anergic, non-responsive or even regulatory T cells are generated by continuous antigen stimulation or the interaction with regulatory immune cells such as tolerogenic DCs, CD4^+^ T regs and CD8^+^ T regs (57–60) (**Figure 2**). Furthermore, chronic stimulation with immunosuppressive cytokines (e.g. IL10) drives effector T cells into anergy. Such anergic T cells might become regulatory T cells with suppressive capabilities (61) ensuring tolerance and preventing autoimmune disease (62). It is important to point out that non-responsive CD8^+^ T cells can become functional again by stimulation of the CD137 receptor (63). Of note, CD137 is increased in inflamed intestinal tissue from Crohn´s disease patients compared to ulcerative colitis patients and control intestinal tissue (64).

One should keep in mind that some subsets of the human microbiota are able to induce inflammatory effector IFNγ^+^ Tc1 cells (56, 65). CD4^+^ T cells, for instance, reactive against antigens of the enteric bacteria can drive IBD (66, 67). Similarly, the animal studies carried by Nancey et al. showed that antigen-specific IFNγ^+^ GrB^+^ Tc1 cells, located in the colon lamina propria, induced relapsing colitis (47). In line with this study, Westendorf and colleagues proved that antigen specific CD8^+^ T cells trigger intestinal inflammation. Hemagglutinin (HA)-specific CD8^+^ T cells were adoptively transferred into immunocompetent transgenic VILLIN-HA mice that expressed a HA antigen under control of the villin promoter specifically located in enterocytes. VILLIN-HA mice developed intestinal inflammation 4 days after adoptive transfer of HA-specific CD8^+^ T cells. The inflammation was restricted to the intestinal epithelium where HA-specific CD8^+^ T cells were found (46). These studies proved that a single antigen can induce colitis in mice. However, the identification of unique antigen specific CD8^+^ T cell(s) responsible for the mucosa damage on IBD patients seems practically impossible given the vast majority of microbiota species located in the gut, not to mention all their plausible-derived antigens.

Taken together, these studies support the role of active cytotoxic Tc1 cells in both initiation and chronic development of IBD.

## Regulatory CD8^+^ T Cells Are More Than Immunosuppressive Cells In IBD

Immune tolerance is a key process for maintaining intestinal homeostasis, not only to itself but also to harmless microbiota and food (6). A break on this tolerance leads to inflammation. Regulatory T cells are believed to play an important role in maintaining homeostatic immunity (e.g. production of IL10 and TGFβ) by limiting pro-inflammatory cytokine responses and by repairing tissue (3, 49). The importance of immunosuppressant CD8^+^ T regs for mucosal healing is supported by their presence in lamina propria of healthy gut (40, 41) (**Figure 2**). However, regulatory T cells are also found in high quantities in the inflamed tissue of IBD patients (40). An elegant study conducted by Boland and colleagues on single cell RNA sequencing (scRNA-seq) found an augmentation of T regs in rectal mucosal tissue of ulcerative colitis patients during active disease (19). Although the study does not discriminate between CD4^+^ or CD8^+^ T regs, due to the single cell sorting on CD45, it makes clear the presence of regulatory T cells at sites of inflammation. Accordingly, classical CD4^+^ Foxp3^+^ T cells are found in active sites of inflammation of IBD (68). Likewise, lamina propria CD8^+^ T regs accumulate in inflamed mucosa of IBD patients in higher frequencies compared to controls (40).

The presence of regulatory T cells at sites of inflammation might either *i*) reflect their attempt to suppress inflammation; *ii*) suggest a dysfunction/impairment of the cells; *iii*) indicate great plasticity in a context-dependent manner and/or *iv*) show diverse populations among T regs (3, 19). At first sight the presence of regulatory T cells at sites of inflammation seems perfectly fitting; one might assume as an attempt to reduce inflammation. Nevertheless, in chronic inflammatory diseases the inflammation is long persisting, raising the possibility that they are, in fact, impaired or not fully functional. In fact, one study found that CD8^+^ T regs might not be properly stimulated by IECs in IBD patients (41). Lamina propria CD8^+^ T regs were decreased in IBD patients and were unable to mediate suppression *in vitro* (41). Furthermore, given the pro-inflammatory environment of chronic inflammation sites in the intestine, the excessive amounts of IFNγ might reprogram CD8^+^ T regs, just as shown with CD4^+^ Foxp3^+^ T regs. Domínguez-Villar et al. showed that CD4^+^ Foxp3^+^ T regs can produce IFNγ when stimulated in a Th1 cytokine environment (69). Even more, CD4^+^ Foxp3^+^ T regs from inflamed tissue of Crohn’s disease patients were able to produce IL17 and IFNγ while retaining suppressive function (70). Whether this scenario applies to CD8^+^ T regs in an IFNγ/TNFα enriched environment remains to be determined, as well as whether or not they aggravate inflammation.

It is worth to mention the ample heterogeneity of T regs. Different subsets of regulatory T cells have been spotted based on their origin, activation status, location and expression molecules (3). For instance, CD28^-^ CD8^+^, CD122^+^ CD8^+^, C39^+^ CD26^-^ CD8^+^, LAG3^+^ Foxp3^+^ CTLA4^+^ CD8^+^, CD103^+^ CD8^+^ and IL10^+^ CCR7^+^ CD45RO^+^ CD8^+^ constitute regulatory CD8^+^ T subsets that exhibited suppressive abilities in transplantation, autoimmunity and IBD (42, 44, 71). Phenotype and function of immunosuppressant CD8^+^ T regs have been studied on the well-accepted immunodeficient RAG2^-/-^ colitis mouse model. RAG2^-/-^ mice are characterized by the lack of mature B and T cell. Colitis is then induced by the intravenous injection of syngeneic CD4^+^ CD45RB^high^ T cells. The adoptive transfer of either CD28^-^ CD8^+^ (44) or CD122^+^ CD8^+^ T regs (42) effectively prevented colitis. Suppression mechanisms of CD28^-^ CD8^+^ and CD122^+^ CD8^+^ T regs are, although not exclusively, the production of IL10 and TGFβ (42, 44). Interestingly, pre-activated CD122^+^ CD8^+^ T regs not only prevented colitis, but also improved well-established disease (42).

CD39^+^ CD8^+^ T cells, associated to a regulatory phenotype, are found within lamina propria under homeostatic conditions and their frequency is decreased in IBD patients compared to healthy controls (32). Similar results were reported by Huang et al. in a pediatric IBD cohort. Furthermore, in a pilot clinical study by the same group, treatment of pediatric colitis and undefined IBD with a cyclic AMP-elevating phosphodiesterase (PDE) inhibitor increased expression of CD39 in intraepithelial T cells and improved clinical symptoms. Hence, CD39^+^ CD8^+^ T cells could be a protective pool by maintaining homeostasis. Interestingly, immunosuppressive and cytotoxic genes are simultaneously expressed in colonic *ENTPD1* (CD39-encoding gene) CD8^+^ T cells (34). Another example of this scenario is the double positive Foxp3^+^ IL17^+^ CD8^+^ T cell pool. Foxp3^+^ IL17^+^ CD8^+^ T cells were found in lamina propria´s inflamed mucosa of IBD patients and were increased in ulcerative colitis compared to Crohn´s disease patients (40). The authors argued though it might be an intermediate population from regulatory T cells towards Tc17, a pro-inflammatory phenotype (**Figure 3**). Remarkably, T cell plasticity can also be modulated by commensals themselves and/or by their metabolites (7). Reduced frequencies of CD4^+^ T regs are found in the colon of germ-free mice and they are less suppressive (72). Comparably, microbiota metabolites impact the frequencies of CD8^+^ T reg subsets (73).

It is important to point out that some regulatory T cell subsets are antigen dependent (74, 75). One might assume that a good fraction of them are food/commensal specific. For instance, CD4^+^ T regs recognize antigens derived from microbiota and food (76) and they are immunoregulatory (77, 78). Similarly, animal models showed the presence of ovalbumin (OVA)-specific CD8^+^ T regs increased in lamina propria when animals were exposed to low OVA antigen dosages overtime (*i.e.* tolerogenic conditions) (79). Moreover, adoptive transfer of OVA-specific Foxp3^+^ CD25^+^ CD62L^low^ CD127^low^ CD4^+^ T regs into refractory Crohn´s disease patients was well tolerated and showed a clinical improvement (78). Interestingly, this OVA-specific T regs produced not only high amounts of IL10, but also granzyme B and IFNγ (78), truly reflecting the complexity of immunoregulation. To the best of our knowledge there are no reports on food-derived antigen specific CD8^+^ T cells in IBD patients, despite the fact that CD8^+^ T cells recognize food-derived antigens (80–82) and that IBD is as well associated to food intolerance (83, 84).

Taken together, these experiments show the importance of different CD8^+^ T reg subsets in regulating gut homeostasis, and that some subsets might be impaired in IBD.

## Other CD8^+^ Subsets Are Involved In IBD

IL17-producing CD8^+^ T cells, or Tc17, are involved in the homeostasis of a healthy gut. They exhibit diminished cytolytic function (e.g. poor expression of granzyme B) and produce high amounts of IL17A, IL-17F and IL22 (23). IL17 and IL22 are relevant cytokines for gut homeostasis by promoting repair and barrier function of the intestinal epithelium, as well as protection against microbiota fungi (3, 85). However, type 17 immunity has been implicated on the pathogenesis of IBD (3, 36, 40, 86, 87). IL-17 levels were increased in the mucosa and serum of IBD patients (87) and Tc17 cells have been revealed as the major source of IL17 in inflamed tissue (36). Correlatively, circulating Tc17 cells are increased in IBD patients compared to controls (36, 40) and Tc17 are expanded in the colon of patients during inflammation (36). These Tc17 cells express genes associated to cytotoxicity as well as increased expression of TNFα (36). Such profile might be explained partially due to the location of these cells. Colonic Tc17 are, indeed, in close proximity to metabolites produced by microbiota, such as short-chain fatty acids (SCFAs). Some SCFAs increased cytotoxic activity of Tc17, e.g. increased expression of IFNγ and granzyme B (73). Given the dysbiosis observed in patients suffering from IBD, the presence of pro-inflammatory Tc17 might be a reflection of an unbalanced microbiota, since under homeostasis SCFAs favor IL10-related immune tolerance (88). Moreover, immunodeficient mouse models have shown the association of Tc17 with the development of severe colitis (89). These CD8^+^ T cells co-express IL17 and IFNγ (89). Double positive IFNγ^+^ IL17^+^ CD8^+^ subsets have been found as well in both blood and inflamed mucosal biopsies of IBD patients (40). This double positive pool could be a distinct cytotoxic subset or an intermediate population from Tc17 towards Tc1 (40, 73, 90). Indeed, one study found *in vitro* polarized Tc17 cells become functional IFNγ^+^-producing Tc1 cells after adoptive transfer in a tumor-bearing mouse model (91).

There is still controversy on accepting Tc22 as a separate lineage since Tc17 can produce IL22 as well (23). Although CD4^+^ Th22 contribute to the chronic inflammation of IBD (92), and Tc22 cells have been correlated to other chronic inflammatory diseases such as psoriasis and atopic dermatitis (93, 94), no reports on Tc22 and the pathogenesis of IBD have been published.

These studies indicate the heterogeneity of the CD8^+^ T cell pool and how the delicate balance between health and chronic disease depends on a variety of factors.

## Intraepithelial And Lamina Propria Compartments

Limited is the data reported on intraepithelial and lamina propria CD8^+^ T cells in health and IBD (95); however, few studies have ventured to provide such information. In homeostatic conditions, CD8^+^ T cell frequencies are slightly higher in lamina propria compared to PBMCs (32) reflecting the need of constant surveillance in the gut. Furthermore, intraepithelial and lamina propria compartments harbor similar CD8^+^ T cell subsets. Additionally, circulating and tissue resident CD8^+^ T subsets locate within the epithelium (30). Certainly, the chronic inflammation observed in IBD has an impact on cell compositions of the mucosa. Patients with Crohn´s disease showed a decreased amount of CD8^+^ T cells within the epithelium at inflamed sites, whereas the CD8^+^ T cell frequency is increased in lamina propria of the non-inflamed tissue. These cells might belong mainly to the CD103^+^ CD8^+^ Trm subset (30) and support the homeostasis of the non-inflamed tissue. How the altered representation of CD8^+^ T cells in diseased tissue correlates to transmural inflammation remains to be investigated.

Besides, diverse CD8^+^ T cell subsets seem to be preferentially located in one or the other compartment. Bottois and colleagues found that the epithelium harbored mainly CD103^+^ CD8^+^ Trm, whereas pro-inflammatory GrB^+^ CD8^+^ T cells were mainly situated in the lamina propria of the inflamed mucosa of Crohn´s disease patients (20). The specific proportions in which different CD8^+^ T cell subsets are found in every compartment, in health and IBD, have not yet been reported.

One might wonder if the function of a given CD8^+^ T cell subset is affected due to their location. In that regard, animal models provide a tool to study the performance of each subset. For instance, both intraepithelial CD28^-^ CD8^+^ and lamina propria CD28^-^ CD8^+^ equally prevented development of colitis in the CD4^+^ CD45RB^high^-induced colitis mouse model (44) indicating that their location might be reflecting their demand not their function. Comparison reports on other CD8^+^ T cell pools, located on either intraepithelium or lamina propria, have yet not been published.

## Is The Memory Of CD8^+^ T Cells Good Or Bad In IBD?

Memory T cells offer a long-lived protective immunity, respond faster after re-exposure to antigen, and accelerate recruitment of circulating immune cells (15). Memory subsets have been identified according to their location and expression of surface molecules: central memory (Tcm, defined as CD45RA^-^ CD27^+^ CD62L^high^), effector memory (Tem, defined as CD45RA^-^ CD27^-^ CD62L^low^) and tissue-resident memory (Trm, defined as CD103^+^) CD8^+^ T cells (96) (**Figure 1**). CD8^+^ Tcm cells are increased in inflamed intestine biopsies of adult ulcerative colitis patients with active disease, whereas CD8^+^ Tem frequency is decreased compared to controls (38). However, in a pediatric IBD cohort with active disease, colonic *GZMK^+^* (granzyme K-encoding gene) CD8^+^ Tem cells were clonally expanded (34), suggesting their active role during inflammation (97).

Since Trm are resident to the gut, more studies have been reported on this memory cell type in IBD. Adult patients in remission had a significant increase on CD103^+^ CD8^+^ Trm cells compared to active disease (35). Furthermore, CD103^+^ CD8^+^ Trm cells were decreased in inflamed tissue from adult IBD compared to non-inflamed biopsies and controls (20, 33, 35, 38). Similarly, Huang et al. reported on the decreased frequencies of colonic *ITGAE* (CD103-encoding gene) CD8^+^ Trm on a pediatric IBD cohort with active disease (34). These findings suggest the regulatory function of Trm. Indeed, CD103^+^ CD8^+^ Trm cells produce IL10 and express regulatory-related markers (e.g. CD73, CD39) (11, 33, 41, 43). Additionally, CD103^+^ CD8^+^ T cells were able to suppress inflammation in mouse models of colitis (11, 43). Hence, CD103^+^ CD8^+^ Trm might be involved in tissue homeostasis and immunosuppressive activities. Unfortunately, due to limited availability of mucosal tissue samples and low cell numbers, direct assessment of their suppressive activity represents a challenge.

It should be highlighted that as any other CD8^+^ pool, Trm show heterogeneity (22), and some of the subsets might be involved in the pathogenesis of IBD. For instance, Bottois et al. showed two different CD8^+^ Trm subsets resident to the ileum of Crohn’s disease patients (20). They could be identified by the almost exclusive expression of either CD103^+^ or KLRG1^+^, both e-cadherin receptors (20, 30). As already described, CD103^+^ CD8^+^ Trm cells were present in the mucosa of both controls and Crohn’s disease patients. However, those from Crohn’s disease patients have an increased expression of pro-inflammatory cytokines, proteases (e.g. granzyme K), and Th17-related genes, suggesting that although they are found in a healthy context, there are molecular differences in the context of inflammation. Furthermore, the authors suggest that CD103^+^ CD8^+^ Trm cells might recruit the KLRG1^+^ CD8^+^ Trm subset into inflamed tissue. KLRG1^+^ CD8^+^ Trm cells are mostly found in the periphery and have a higher cytotoxic potential evidenced by the intracellular levels of granzyme B. In accordance, KLRG1^+^ CD8^+^ Trm cells were increased in inflamed tissue of Crohn’s disease patients compared to non-inflamed and controls (20). These results could not be confirmed by Jaeger and collaborators, who found no difference in mucosal KLRG1^+^ CD8^+^ Trm cell frequencies between Crohn´s disease patients with severe disease activity and controls (30). Such discrepancy might rely on disease/clinical status, treatment, duration of the inflammation, age of the patients or even tissue sampling (e.g. colon, ileum). Additionally, results on CD103^+^ CD8^+^ Trm published by Bottois et al. and Roosenboom et al. contradicted the findings by Zundler and colleagues (20, 35, 37). Zundler et al. found increased frequencies of CD103^+^ CD8^+^ Trm in lamina propria of IBD patients (37). However, this population was as well characterized by the presence of the T cell activation and retention marker, CD69^+^. Even more, IBD patients with high frequencies of CD103^+^ CD69^+^ CD8^+^ Trm had a worse outcome of the disease over time (37) demonstrating how diverse in phenotype and function Trm cells could be. Another tempting CD8^+^ Trm subset possibly involved in the pathogenesis of IBD is the one identified by Boland et al., the Eomes^hi^ CD8^+^ Trm cells. These cells were enriched in inflamed tissue of ulcerative colitis patients and showed enhanced inflammatory properties (19). It is plausible that depending on the cytokine context, Trm subsets are either pro- or anti-inflammatory (33, 37). They have different locations within the intestinal mucosa, hence different potential cellular partners (20); thereafter, a different outcome could be expected in the plasticity of the cells.

Furthermore, Trm are commonly believed to be non-circulating effector memory cells and to reside within tissue (37, 96). However, some Trm clones are able to exit intestinal tissue and recirculate (98). Noble et al. found circulating CD8^+^ Trm in peripheral blood of IBD patients and they were responsive to intestinal bacteria (33). This might explain, in part, why in some cases of IBD inflammation can be observed in tissue outside the gastrointestinal tract (3, 19).

## Where Do Pro-Inflammatory CD8^+^ TRM Cells Come From?

An important pool of memory CD8^+^ T cells are derived from Tc1 cells, demonstrated by their ability to produce IFNγ upon antigen reencounter. Evidence suggests that some memory cells are derived from Tc17 lineage as well, but it is not clear whether other CD8^+^ T cell subsets wellspring memory cells (23). It seems reasonable that in homeostatic conditions (*i.e.* healthy gut) Trm are derived from those responsible for maintaining homeostasis, whereas in autoimmune diseases and chronic inflammatory diseases, Trm are derived from those producing the damage (99). In IBD, the same pro-inflammatory Tc1 and Tc17 might be the ones offspringing pro-inflammatory Trm (**Figure 3**). It seems that at least a portion of Trm cells are antigen specific as exogenous stimulation peptide is sufficient to induce cytokine expression and cytolytic molecules (100). Making pro-inflammatory Trm an ally and rendering them tolerogenic to antigens IBD patients react to (e.g. by continuous antigen exposure) might be a possible therapeutic approach.

These studies suggest diversity among Trm and their impact on the chronic inflammation of IBD. Trm might be immunoregulatory in a healthy gut, but Trm found in gut of IBD patients might be leaning towards a pro-inflammatory phenotype regardless of the amount found in tissue. Moreover, in order to recognize diverse Trm pools, cells should be better characterized by surface (e.g. CD103, CD69, CD49a, CD101, CXCR6) and intranuclear markers (e.g. Hobit, Runx3, Blimp-1) (37) in future studies.

## Exhaustion Of CD8^+^ T Cells Might Be Benefitial In The Gut

T cell exhaustion is a stage where T cells are chronically stimulated by antigens, effector functions are reduced (e.g. ability to secrete cytokines) and the expression of inhibitory receptors is upregulated (101). That these less responsive exhausted T cells persist during chronic antigen presentation might also reflect their nature to partially prevent immunopathology (101). Accordingly, exhausted expression signature of CD8^+^ T cells correlated to a milder disease course and better prognosis (21, 29, 102, 103).

CD8^+^ T cell exhaustion transcriptional signatures revealed by single-cell RNA sequencing are unique (21, 101). Corridoni et al. identified chronically activated Tc17, IL26^+^ and granzyme K^+^ CD8^+^ T cells in the mucosa of ulcerative colitis patients showing features of ”exhaustion”. Furthermore, some subsets, for instance, IL26^+^ CD8^+^ exhausted T cells exhibit a regulatory phenotype suggesting its ability to reduce inflammation (21). Since exhausted CD8^+^ T cells are less responsive to microbiota-derived antigens, their existence in mucosal tissue might contribute to homeostasis. Thereafter, the adoptive transfer of highly suppressive “exhausted” CD8^+^ T regs into IBD recipients might be a treatment worth to explore.

## Immunosupressant CD8^+^ T Regs As A Therapeutic Approach In Ibd

There is no doubt that regulatory T cells represent an attention-grabbing strategy for reaching homeostasis in chronic inflammatory diseases. However, regulatory T cells cannot be isolated in sufficient numbers, let alone those specific to self-, microbiota-, or food-derived antigens. Engineering of T cells carrying a synthetic receptor, the so-called chimeric antigen receptor (CAR), has been proven with optimistic results, mainly in treatment of cancer. Briefly, autologous CD8^+^ T cells obtained by leukapheresis are enriched and engineered to express a specific recombinant receptor that recognizes a given antigen expressed on tumor cells (104). Viral and non-viral vectors can be used for gene transduction. Afterwards CAR T cells are expanded *ex vivo* and the non-/poorly-engineered T cells are depleted by CliniMACS Prodigy, an automated good manufacturing practice (GMP)-compliant (104, 105). The resultant CAR T cells are re-infused into patients where they lyse tumor cells that express the specific antigen the newly CAR T cell were engineered for (104). It is important to point out that some CAR immunotherapeutic approaches have been GMP-approved as an anticancer immunotherapy (105, 106).

CAR approach has also been tested on autoimmune diseases at responding to self-antigens and re-establishing tolerance (107, 108). In this approach, T cells not only have engineered-CARs but also reprogrammed phenotype by Foxp3 transduction resulting in the conversion of effector cells towards immunoregulatory cells (107, 108). As a proof of concept, Tenspolde and colleagues, engineered CAR-CD4^+^ T cells towards insulin, and from effector towards T reg by Foxp3 transduction (108). These cells showed stable suppressive function, and lived long *in vivo* (108). Similarly, in an immunocompetent mouse model of colitis expressing the carcinoembryonic antigen (CEA) in the small intestine, CEA-specific CAR CD4^+^ T regs inhibited colitis triggered by CEA-specific CAR CD4^+^ effector T cells (107). Two key messages are highlighted. First, in this model, CEA-specific CAR CD4^+^ effector T cells significantly destroyed the epithelial barrier on mice that express CEA in the small intestine leading to colitis; hence, effector cells were triggered by a specific antigen presented on the epithelial cells. Second CEA-specific CAR CD4^+^ T regs were highly immunosuppressant of their effector counterparts (107). Converting CD8^+^ effector cells from IBD patients into CD8^+^ T regs and engineered with CARs that recognize specific antigens expressed in microbiota, food or even autoantigens might be an option worth exploring in IBD. The clinical implications of generating such CAR-T regulatory cells relies on the fact that some self-antigens are elevated in patients with IBD, such as CEA (109), anti-*Saccharomyces cerevisiae* antibody (ASCA) (5) and perinuclear anti-neutrophil cytoplasmatic antibody (pANCA) (110). Of note, pANCA cross-react with antigens found in bacteria‘s outer membrane, implicating microbiota proteins as targets by effector cells (111). Clearly, the election of targeted antigens should be carefully considered, as well as T cell plasticity. In this regard, predisposed phenotype of insulin-specific CAR/Foxp3^+^ transduced T regs remained stable even four months after injection in the non-obese diabetic mouse model (108). However, longer follow-up for survival and immunoregulatory function of engineered antigen-specific CAR T regs is highly advisable. Although it is true that the latest studies described engineering of CD4^+^ regulatory T cells, CAR technology could also be applied to CD8^+^ T cells, since it appears that Tc1 cells are responsible for initiating epithelial barrier damage (46, 47).

Another plausible way of generating tolerant CD8^+^ T regs is by continuous antigen exposure. Mahic and colleagues co-cultured effector CD8^+^ CD25^-^ T cells with CD14^+^ monocytes in the presence of staphylococcal enterotoxin B (SEB) for four days resulting in the generation of regulatory Foxp3^+^ CD25^+^ CD8^+^ T cells (57). Such conditions mimic, in a very artificial way, the intestinal environment where antigens are continuously presented to T cells by APCs. The resultant *de novo* generated CD8^+^ T regs were able to suppress effector CD4^+^ and CD8^+^ T cells by cell contact. Noteworthy, *de novo* generated CD8^+^ T regs express not only IL10, but also granzymes and perforin in high levels (57).

These studies show that “reassigned” antigen specific CD8^+^ T regs, by CAR technology or continuous antigen stimulation, might be a therapy worth investigating.

## Discussion

IBD is a complex disease involving aberrant immune responses. CD4^+^ T cells have long been proven to play a role in the initiation and progression of inflammatory colitis. Not less important, CD8^+^ T cells have been investigated in recent years in more detail to decipher their role on the pathogenesis of IBD. New approaches such as epigenetics, scRNA-seq, and mass cytometry (CyTOF) are identifying CD8^+^ T associated signatures in IBD (19, 21, 30, 34, 36), their intrinsic features, as well as different existing pools. Molecular and cellular profiles of CD8^+^ T cells from both adult and pediatric IBD patients show promising results towards a more personalize therapy, but they still need validation in independent cohorts (5, 18, 29).

Growing data on the diversity and complexity of subsets and stages of CD8^+^ T cells in IBD are now demonstrating their involvement in the course of the disease, either by promoting or by suppressing inflammation. From this point of view, future studies need better characterization of targeted CD8^+^ T cells with more accurate markers in order to elucidate their complex phenotype and functions. Promising new reports shine light in this regard by identifying specific phenotypes in CD8^+^ T cells from IBD patients and segregating them into clusters depending on the RNA/protein expression (19–21). A significant obstacle in differentiating pro-inflammatory *versus* anti-inflammatory CD8^+^ T cells, though, is the limited availability of intestinal tissue from patients as well as from healthy controls. Of note, until now healthy control tissue has consisted of non-IBD patients undergoing bowel resection (e.g. colon cancer, colonic polyposis). Ethical concerns are raised in obtaining healthy tissue from healthy donors given the risks associated with any surgical procedure.

Despite the fact that both Crohn´s disease and ulcerative colitis present chronic inflammation mainly in the gastrointestinal tract, they have distinctive T cell expressions. Crohn´s disease has been associated with a CD4^+^ Th1/CD4^+^ Th17 phenotype, whereas ulcerative colitis leans towards a CD4^+^ Th2 (112). Although the same scenario is seen on CD8^+^ Tc1 in Crohn´s disease *versus* ulcerative colitis (40), patients suffering from ulcerative colitis have a higher frequency of Tc17 and Foxp3^+^ IL17^+^ CD8^+^ T cells in lamina propria compared to Crohn´s disease patients (40). The difference in CD4^+^ pools is supported by the unique intestinal microenvironment (112), but the fact that the same signatures are not reflected on CD8^+^ T cell subsets evidences the complexity of adaptive immune cells.

Highly relevant is the symbiotic relationship between microbiota, its host, and its establishment in order to achieve homeostatic immunity. The break of this balance, the dysbiosis observed in IBD, has a major role in the progression of the disease. Yet, little is known about antigen specific CD8^+^ T cells reacting towards gut microbiota, food or even self-antigens. CD8^+^ T cells found in the peripheral blood of healthy people are immunosuppressant in response to commensal-bacteria found in the skin (8). However, investigation is still needed to determine if the regulatory CD8^+^ T cell response is comparable to microbiota-derived antigens in the lumen of the gut.

Lastly, current treatment options for IBD are immunosuppressive rather than curative, and patients endure lifelong medication that often leads to secondary failures and/or side effects. More effective and precise therapies are needed whether from fecal microbiota transplant, oral anti-IL12 (113), or cellular therapy involving, for example, genetically engineered immunosuppressive T cells targeting antigens from microbiota, food and/or self. Carefully selecting targeting antigens for CAR-Foxp3 T regulatory approach would be a more personalized therapy. IBD patients’ loss of tolerance to self- and microbiota antigens occurs in different degrees resulting in patient subgroups responding differently to selected antigens (114). Generating CD8^+^ T regulatory cells capable of diminishing inflammation in a personalized manner might reduce the risk of adverse side effects.

The delicate balance that holds intestinal homeostasis seems to be compromised in IBD. One of the cellular participants in this grenade field are CD8^+^ T subsets. Deep understanding of the diverse CD8^+^ pools as well as their function might help us to restore the equilibrium, and health of patients suffering from IBD.

## Author Contributions

RCG conceptualized and wrote the manuscript. JD critically revised the manuscript for important intellectual content. All authors contributed to the article and approved the submitted version.

## Funding

RCG was supported by the FORUN Research Program of the Rostock University Medical Center (FORUN project no. 889023).

## Conflict of Interest

The authors declare that the research was conducted in the absence of any commercial or financial relationships that could be construed as a potential conflict of interest.

## Publisher’s Note

All claims expressed in this article are solely those of the authors and do not necessarily represent those of their affiliated organizations, or those of the publisher, the editors and the reviewers. Any product that may be evaluated in this article, or claim that may be made by its manufacturer, is not guaranteed or endorsed by the publisher.
